# Civil disorder, authority credibility and public health: Chile’s unique sociopolitical context in dealing with COVID-19

**DOI:** 10.7189/jogh.11.03019

**Published:** 2021-01-16

**Authors:** Alejandra Caqueo-Urízar, Alfonso Urzúa, Diego Aragón, Diego Atencio, Akaninyene Otu, Sanni Yaya

**Affiliations:** 1 Instituto de Alta Investigación, Universidad de Tarapacá, Arica, Chile; 2 Escuela de Psicología, Universidad Católica del Norte, Antofagasta, Chile; 3 Escuela de Medicina, Universidad de Valparaíso, Valparaíso, Chile; 4 Centro Justicia Educacional CJE, Pontificia Universidad Católica de Chile, Santiago, Chile; 5 Leeds Teaching Hospitals, National Health Service Trust, Leeds, UK; 6 School of International Development and Global Studies, University of Ottawa, Ottawa, Ontario, Canada; 7 Imperial College London, London, United Kingdom

## SOCIAL UNREST AND ITS INTERSECTION WITH COVID-19

On 3 March 2020, the first case of coronavirus was confirmed in the country, approximately five months after 18 October 2019, one of Chile’s biggest episodes of civil unrest since its return to democracy in the 90s [1]. This social unrest was triggered by deep social inequalities and resulted in massive protests throughout the country. The citizens were responding to widespread inequalities in various sectors such as jobs, housing, education and public health and the main Chilean governmental institutions were called to account [2]. These social unrests severely dented the government’s credibility and compromised the reliability of key governmental institutions such as law enforcement and the military, all of them, now playing a key role in the Public Health response to the novel coronavirus.

The perennial inequalities in social rights that have had deleterious effects on the quality of life of the most vulnerable groups in Chile, gave rise to the neoliberal model, developed during the Dictatorship in 1973. Although this model positioned the country as one of the fastest growing economies in the continent, it also led to a deep social discontent, especially among Chile’s lower and middle class. These groups have been advocating for reforms in the minimum wage, pension funds, public health, access and cost of higher education in addition to achieving legitimate rights to public goods such as water, improved public transportation and ending corporate collusion and tax evasion [2].

It is in this complex sociopolitical milieu that the COVID-19 pandemic reached Chile. Since 3 March 2020, COVID-19 has magnified profound social gaps in the country and disproportionately affected the low- and middle-class workers, who account for the great majority of the Chilean workforce. Due to their lean financial resources these workers have not been able to afford the luxury of physical distancing strategies such as remaining at home and have had to go to work every day. Given that this group is likely to function in frontline roles as cleaners, public transport drivers, carer, and health workers, this higher exposure risk to COVID-19 is likely to be contributing to their disproportionate affectation by COVID-19. Key governmental institutions such as the public health sector, economic agencies and law enforcement bodies have been called in to steer the Chilean response to COVID-19. However, the credibility of these institutions has been severely damaged by the preceding wave of social unrests and this has undermined their capacity to mount a robust response to the existential threat posed by COVID-19.

## THE DELEGITIMIZATION OF AUTHORITIES AND INSTITUTIONS IN CHILE: ORIGIN OF THE FRAGILE SITUATION

Given the scenario outlined above, there is palpable mistrust of the main authorities in Chile such as law enforcement, army, senate, the justice system, political parties and Catholic Church [3]. This mistrust has steadily grown over the years and gone from a diffuse and generalized social distrust, to a more explicit and active one in recent years [4]. The deep distrust the political regime and main institutions is further fueled by scandalous cases of corruption that have become publicly known over time (such as PENTA, SQM, CAVAL, MILICOGATE) but have mostly gone unpunished [5]. Another precipitating factor is the low participation of citizens in exercising their franchise during the 2017 elections [6]. Out of a total of 14.83 million eligible voters, only 46% of them voted in the 2017 presidential election [7].

According to the 2018 National Transparency Study, 61% of the respondents drawn from across the nation felt that the current political parties were too corrupt [8]. This corruption is likely to have had a negative impact on democratic processes and further increased the inequalities within the country [9].

Despite the perceived issues with corruption, Chile has a Gini coefficient of 47.7 [10] which is considerably higher than that of other democracies [9]. Although the Chilean economy is one of the fastest growing in Latin America, income inequality remains high and more than 30% of the citizens are economically vulnerable [11].

## THE PUBLIC HEALTH RESPONSE TO COVID-19 IN CHILE: CHALLENGES AND PROSPECTS

The Chilean health care system is divided into private and public sectors; this is based on a mandatory contribution and voluntary private health insurance [12]. While the public sector caters for approximately 75% of the population which constitute the most vulnerable groups [13]; the private sector covers the remaining 25% who can afford to pay voluntary premiums. Due to the unequal life conditions of the users of the public and private sectors, people using the public sector are more likely to get sick than the private sector users [14]. Additionally, out-of-pocket expenditure on health care in Chile is high when compared to other countries [15]. Given this context, the Chilean health system is organized in a way that is clearly segmented by risk and income thus promoting inequity and failing promote access to health care as a constitutional right [12,14,16].

**Figure:**
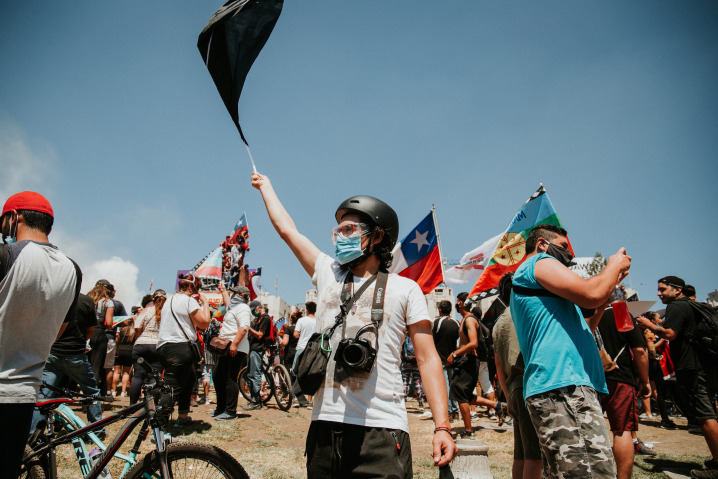
Photo: Photograph taken by Karla Riveros Riveros in the framework of the demonstrations held one year after the social outbreak in Chile. Plaza Baquedano, Santiago, Chile, 18 October 2020. Image granted to the Centro de Justicia Educacional (CJE).

It is under these complex sociopolitical circumstances that the discredited authorities are generating and implementing a public health response to the COVID-19 pandemic. The main strategy of the government is to implement large scale COVID-19 testing coupled with quarantine measures to active COVID-19 cases [17]. In addition to this, a *novel* strategy, based on small lockdowns and dynamic quarantines, was proposed by the already highly questioned government. This strategy was mostly implemented at the capital of Chile, Santiago. This city is divided into 32 administrative units, called *communes*. A dynamic quarantine then, was defined as lockdown of certain communes, based on the number of active cases in that area. These quarantines were then reassessed weekly and lifted or prolonged based on the state of the outbreak in that specific commune. However, decision taking and cut off points were highly dependent on the Ministry of Health, and the methods to determine such measures were not publicly available.

This strategy was built on the premise that Chile has the capacity to test and diagnose most COVID-19 infected individuals, so that they can be properly isolated. This way, the economy of the country is protected from the damaging effects of imposing a nationwide lock down. Under these circumstances the low- and middle-income workers who continue to go to work have a higher exposure risk for COVID-19 compared to the wealthy who can afford to properly isolate at home during this period. This inherent inequality further disadvantages the already vulnerable low- and middle-income class.

Another contentious issue involved the patients who have recovered from COVID-19. Although Chile has a fairly low mortality rate and one of highest recovery rates from COVID-19 worldwide [18], it has been stated by the government that the recovered cases are *estimations* and it is unclear whether these *recovered* patients have actually cleared the infection and are no longer an infectious risk [19].

Despite the imposition of dynamic quarantines and partial confinement in an attempt to preserve the national economy, the financial situation appears to have worsened with increasing unemployment. Although the dynamic quarantine approach did show to have an effect on lowering mobility and viral transmission [20], it was insufficient to reach a relative control of the outbreak and so a total quarantine was imposed for Santiago on May 15 2020 [21,22].

The economic crisis that has ensued led opposition parliamentarians to present a bill that would allow the withdrawal of 10% of the workers' pension savings [23]. The government opposed this move and purposed bonds and loans for the middle class instead. It is important to note that the middle class has been historically the least supported class by state benefits, and thus, such proposals were not well perceived by the middle-class citizens [24,25]. In addition, the requirements to access these benefits were prohibitive, which meant that many citizens were not considered as beneficiaries of these benefits and the population did not see them as an acceptable option. Finally, the voluntary withdrawal of 10% of the pension funds was approved, and the government, faced with waning popularity, opted to sign and ratify the law [26].

These complex issues have combined to make the Chilean response to COVID-19 incredibly unique and challenging task. Some of the measures adopted by the government have not been favorably received by various sections of the community. As the debates continue to rage on the safest and most acceptable approaches to tackling COVID-19 in Chile, the number of COVID-19 cases continue to rise, and the preexisting inequalities continue to grow. The COVID-19 pandemic may be the opportunity for Chile to begin to address the yawning gap in the socio-economic determinants of health such as education, housing, income and access to public goods and services.
